# Polycomb group gene BMI1 controls invasion of medulloblastoma cells and inhibits BMP-regulated cell adhesion

**DOI:** 10.1186/2051-5960-2-10

**Published:** 2014-01-24

**Authors:** Ashirwad Merve, Adrian M Dubuc, Xinyu Zhang, Marc Remke, Patricia A Baxter, Xiao-Nan Li, Michael D Taylor, Silvia Marino

**Affiliations:** 1 Blizard Institute, Barts and The London School of Medicine and Dentistry, Queen Mary University of London, 4 Newark Street, London E1 2AT, UK; 2 Program in Developmental & Stem Cell Biology, The Hospital for Sick Children, 101 College Street, TMDT-11-401M, Toronto, ON M5G 1L7, Canada; 3 Department of Laboratory Medicine & Pathobiology, University of Toronto, Medical Science Building, 1 King’s College Circle, 6th Floor, Toronto, ON M5S 1A8, Canada; 4 Department of Surgery, Division of Neurosurgery, The Hospital for Sick Children, 555 University Avenue, Hill 1503, Toronto, ON M5G 1X8, Canada; 5 Texas Children’s Cancer Center, Texas Children’s Hospital, Baylor College of Medicine, 6621 Fannin Street, MC-3-3320, Houston, TX 77479, USA

**Keywords:** BMI1, BMP, Medulloblastoma, Cell adhesion, Cell motility

## Abstract

**Background:**

Medulloblastoma is the most common intracranial childhood malignancy and a genetically heterogeneous disease. Despite recent advances, current therapeutic approaches are still associated with high morbidity and mortality. Recent molecular profiling has suggested the stratification of medulloblastoma from one single disease into four distinct subgroups namely: WNT Group (best prognosis), SHH Group (intermediate prognosis), Group 3 (worst prognosis) and Group 4 (intermediate prognosis). BMI1 is a Polycomb group repressor complex gene overexpressed across medulloblastoma subgroups but most significantly in Group 4 tumours. Bone morphogenetic proteins are morphogens belonging to TGF-β superfamily of growth factors, known to inhibit medulloblastoma cell proliferation and induce apoptosis.

**Results:**

Here we demonstrate that human medulloblastoma of Group 4 characterised by the greatest overexpression of BMI1, also display deregulation of cell adhesion molecules. We show that BMI1 controls intraparenchymal invasion in a novel xenograft model of human MB of Group 4, while *in vitro* assays highlight that cell adhesion and motility are controlled by BMI1 in a BMP dependent manner.

**Conclusions:**

BMI1 controls MB cell migration and invasion through repression of the BMP pathway, raising the possibility that BMI1 could be used as a biomarker to identify groups of patients who may benefit from a treatment with BMP agonists.

## Background

Medulloblastoma (MB) is a highly aggressive embryonic tumour of the cerebellum, and the most common paediatric malignant brain tumour. Despite significant advances in treatment, MBs are still associated with significant mortality and high morbidity. Current therapeutic intervention involves maximum surgical resection, cranio-spinal irradiation and dose-intensive chemotherapy, which often leads to severe secondary disabilities among the survivors and, importantly, does not take into account the specific molecular mechanisms driving tumour growth. Improved risk stratification of patients prior to therapy in addition to novel molecularly tailored drugs are therefore urgently needed to improve the prognosis of children with MB.

Recently, genome-wide expression analysis has significantly advanced our understanding of the molecular pathogenesis of MB, identifying four distinct molecular subgroups affecting prognosis and predicting response to therapy [1-4]. Two groups, characterized by activation of WNT and Sonic Hedgehog (SHH) pathways respectively, have been thoroughly characterized, while the molecular signatures underlining Groups 3 and 4 are less well defined. WNT subgroup tumours have the best prognosis and while Group 3 represent the most malignant molecular variant, associated with the worst patient outcome, both SHH Group and Group 4 represents subgroups with an intermediate prognosis [5]. Metastatic disease, characterized by leptomeningeal spread and dissemination via the cerebrospinal fluid, is an important, independent adverse prognostic factor, present in up to 35% of patients at the time of diagnosis [6]. Higher incidence of metastatic disease is found among MB of Groups 3 and 4 and it contributes to their poor prognosis [5].

Cerebellar development is guided by a complex network of molecular and cellular mechanisms critical for embryonic and postnatal development, while deregulation of these pathways plays an essential role in MB formation [7]. *BMI1* is a potent inducer of neural stem cell self-renewal and neural progenitor cell proliferation during development and in adult tissue homeostasis. *BMI1* overexpression is observed in numerous human cancers, including MB [8]. We recently reported that *BMI1* is most highly expressed in Group 4 MB, a molecular group with the lowest expression levels of *TP53 *[9]. In support of these findings, overexpression of *BMI1* with concomitant *Tp53* loss in the granule cell lineage induces MB formation, albeit at very low frequency [9].

Bone morphogenetic proteins (BMPs) of the transforming growth factor-β (TGF-β) superfamily are negative regulators of cell proliferation and cell survival in the developing brain [10]. Activated BMP receptors (BMPR) phosphorylate Smad1, Smad5 and Smad8 proteins, which in turn results in Smad4 nuclear translocation, where it acts as a transcriptional regulator [11]. During cerebellar development, BMP2 and BMP4 inhibit SHH-induced granule cell progenitors (GCPs) proliferation *in vitro*, resulting in differentiation, whereas BMP7 has the opposite effect [12,13]. BMP signalling remains intact in MB cells [13] and exogenous BMP2 induces apoptosis in a dose and time dependent fashion in primary human MB cells [14,15]. Moreover, BMP2 inducing agents such as retinoic acid have been shown to reduce MB tumour growth *in vitro* and *in vivo *[16].

Recently, we demonstrated in a genetically engineered mouse model that BMI1 controls cellular interactions between granule and glial progenitors during cerebellar development through repression of the BMP pathway [17].

In this study, we use a novel xenograft model of Group 4 MB and *in vitro* assays to assess the implications of this novel molecular connection for MB pathogenesis.

## Methods

### MB cell lines and primary cells

MB cell lines (UW228-2, D-425, D-458, D-341 and DAOY) were obtained from ATCC. DAOY and D-458 were used for functional studies: DAOY were grown as adhesive monolayer while D458 were grown in suspension. Both cells lines were cultured and maintained in Improved MEM media (Gibco) containing L-lysine and Glutamate, supplemented with 10% FBS (Gibco), Penicillin (Sigma) 10 U/ml and Streptomycin (Sigma) 0.01 mg/ml. For passaging, DAOY cells were trypsinised with 1% Trypsin EDTA (Gibco).

Primary human MB cells (ICb-1299) were obtained from Dr Xiao-Nan Li, Baylor College of Medicine, Texas Children’s Cancer Centre, USA. These cells were originally isolated from an anaplastic MB, stage M3 and maintained as intracerebellar xenografts in mice after orthotopic transplantation of fresh tumour [18]. Genetic profiling of the original tumour and primary cells classified them as Group 4 MB [19]. For expansion and knock down studies, these cells were cultured in Dulbecco’s Modified Eagle Medium (D-MEM) with high glucose (Gibco) supplemented with 10% FBS (Gibco), Penicillin (Sigma) 10 U/ml and Streptomycin (Sigma) 0.01 mg/ml.

### MB gene expression profiling and pathway analysis

Transcriptional profiling of BMI1kd versus wild-type MB cell lines (DAOY) on Affymetrix Gene Chip Genome 133 2.0 Plus Expression arrays were downloaded from Gene Expression Omnibus (GSE7578). Similarly, human primary MB expression data across a 285 tumours previously profiled on Affymetrix Human Gene 1.1ST arrays were downloaded from GSE37382. All CEL files were analysed using Affymetrix Expression Console (Version 1.1) as previously described in Northcott et al. [3]. Genome-wide statistically significant differences in gene expression patterns were calculated using the Wilcoxon Rank Sum Test with Benjamini-Hochberg FDR correction (q < 0.01) in MultiExperiment Viewer (MeV). Statistically significant gene sets were further filtered on the basis of absolute fold-changes greater or equal to 1.5. Pathway analysis was performed using GSEA Molecular Signature Database (MSigDB) using the curated pathways described, and an FDR q-value below 0.05. Unsupervised hierarchical clustering of BMI1-high, TP53-low versus BMI1-low, TP53-low Group 4 medulloblastomas was performed using the top 1500 genes with the highest standard deviation using the Pearson Correlation metric and bootstrapping as described previously [3].

### RNA interference

BMI1 knock down (BMI1kd) was achieved either by means of siRNA (transient) or shRNA (stable) technology. For transient BMI1kd, FlexiTube siRNA (Qiagen) specific for BMI1 (containing a mix of Hs BMI1 1, Hs BMI1 2 and Hs PCGF4 3) was used. All Stars Negative siRNA (Qiagen), referred to as scrambled (Scr) was used as control. 70-80% confluent DAOY or D-458 cells were treated with siRNA at a final concentration of 30nM in combination with HiPerFect Transfection Reagent (Qiagen) according to manufacturer’s protocol. The transfected cells were incubated for 48 hr prior to functional studies for best knock down efficiency, as assessed by Western blot and qRT-PCR analysis. For stable BMI1kd, human GIPZ lentiviral shRNAmir BMI1 construct (Open Biosystems) containing a CMV-driven GFP reporter and seven clones of target sequences of human Hs BMI1 (NM_005180) was used. The plasmids (human GIPZ shRNAmir BMI1 construct, pGIPZ empty vector, HIV1 and VSVG) were first purified using QIAfilter maxikit (Qiagen), then packaged using HEK293T cells to produce lentiviral viruses with a final titre of 2.5 – 11 × 10^8^ TU/ml. Scr vectors were packaged with pGIPZ empty transfer vector, as described above. DAOY and ICb1299 cells were infected after mechanical dissociation at a multiplicity of infection (MOI) of 12.5 and 25 respectively, incubated for 72 hr and FACS sorted for GFP prior to further culture. The efficacy of knock down was assessed by western blot and qRT-PCR analysis at multiple time points after passaging. BMI1 knock down studies on DAOY and D-458 MB cell lines to investigate BMP pathway activation by immunofluorescence and to demonstrate cell aggregate formation were performed using siRNA method, all other experiments were conducted with a lentiviral mediated shRNA method. All experiments were conducted at least in triplicates.

### BMP pathway activation and inhibition

To induce BMP pathway, recombinant human BMP-4 (R&D systems) was used to treat the cells at a concentration of 100 ng/ml for 24 – 36 h. To inhibit the BMP pathway, mouse recombinant Noggin/Fc Chimera (Sigma) was added to the cultures at a concentration of 1 μg/ml and the cells were incubated for a minimum of 24 h prior to functional analysis. When required, BMI1 kd was carried out concomitantly as previously described.

### Western blotting and qRT-PCR

Total protein were extracted from the cell pellets with RIPA buffer, Tris HCL, NaCl, 1% NP40, 0.5% sodium deoxycholate, 0.1% SDS and protease inhibitors and sonicated. 25 μg of protein homogenates were separated by acrylamide gel electrophoresis along with protein standard ladder (Bio-Rad), transferred onto nitrocellulose membrane by further electrophoresis, according to standard protocols. The membrane was pre-incubated with 5% w/v milk solution for 1 hr, followed by incubation with primary antibodies, either mouse monoclonal anti-BMI1 (Millipore) 1:500, rabbit polyclonal anti-pSMAD1,5,8 (Cell Signalling) 1:1000, rabbit polyclonal anti-SMAD1,5,8 (Sant Cruz Biotechnology) 1:400 or mouse monoclonal anti-alpha-tubulin antibody (Sigma) 1:5000. Appropriate secondary antibodies, ECL peroxidase labelled anti-mouse antibody (Millipore) 1: 3000, horse radish peroxidase anti-rabbit IgG (Santa Cruz Biotechnology) 1:3000 were used for detection, followed by detection of HRP using Enhanced Chemoluminiscence substrate (Thermo Scientific).

Total RNA was extracted from the cell pellets using RNeasy microkit (Qiagen). Reverse Transcription was carried out using Quantitect kit (Qiagen) and triplicates of cDNA templates were subjected to TaqMan gene expression analysis according to standard protocols (BMI1 primer Hs00180411_m1 and housekeeping gene beta-actin primer Hs99999903_m1, Applied Biosystems run on a SDS 7500 system).

### In vitro migration assays

Transwell migration assay: This assay was performed as per published protocols [20,21]. Transwell® inserts (24 mm diameter with pore size of 8 μm, Corning) were first coated with basement membrane or ECM substrates - Matrigel 100 μg/ml (BD Biosciences) or Type I Collagen (First Link) 20 μg/ml. The coating procedure was performed as per the manufacturer’s protocol, and were left overnight at 37°C for adequate coating after which the excess extract solution was carefully removed. A constant number of cells (4 × 10^5^ cells/well in 2 ml serum free media) were incubated on the top surface of these inserts placed in culture plate chambers. Media containing 10% serum (chemoattractant) was added to the bottom of the chamber. After incubating for 12 hr, the cells in the inserts were fixed using 4% PFA and stained with Gill’s Hematoxylin. Non-migrated cells from the top surface of the insert membrane were scraped, preserving only the migrated cells on the bottom part of the membrane. Nuclei of migrated cells were counted in 5 random 20X fields in each membrane using ImageJ software. The values were expressed as mean ± SD. All experiments were conducted in triplicates.

Gap closure assay [21]: A constant number of cells (0.5 × 10^5^ cells/well) were plated in a 24-well plate without ECM substrate until they reached confluence. A wound was incited (using a 20 μl pipette tip) in each well by removing ~80 μm wide strip of cells. The wounded monolayer was washed with medium to remove floating cells. The cells were incubated in time lapse chamber (37°C and 5% CO_2_) and image acquired (using Nikon inverted microscope, 20X lens) every hour, for 12 hr. Three random areas for each well were imaged, and 3 set of wells were analysed for each condition tested. The images were compiled and a movie was created using Metamorph software (Molecular Devices, Sunnyvale, California). The area of gap closure was measured as mean ± SD. All experiments were conducted in triplicates.

Individual cell motility assays: The assay was performed as per published protocols [22]. Ten cells in each well were tracked by means of Metamorph software (Molecular Devices, Sunnyvale, California) using image acquired from time lapse microscopy (Nikon) and the distance of migration was calculated and expressed as mean ± SD. The distances were compared with controls. The experiments were conducted in triplicates.

### Analysis of proliferation and apoptosis

The CyQUANT® NF proliferation assay kit (Invitrogen) was used. The cells were plated in Costar® 96 well plate (Corning Inc.) at a constant density of 10^3^ cells per well in 100 μl medium and were incubated at 37°C overnight to achieve cell adherence. Initially, 1X Dye Binding solution was prepared by mixing 1X Hank’s balanced salt solution (HBSS buffer) with Dye Reagent (containing digitonin and dimethylsulfoxide), as per manufacturers protocol. The medium was then removed and replaced by 100 μl of 1X Dye Binding solution in each well. The plate was incubated at room temperature for 30 min and the fluorescence intensity (excitation set at 480 ±10 nm, and emission detection at 530 ± 10 nm) of each sample was measured by Synergy HT microplate reader (BioTek) using KC4™ v3.4 software (BioTek). Three independent experiments with three technical replicas each were performed.

In addition, the proliferation capacity was also assessed by means of growth curve analysis. The DAOY cells (100,000) were seeded in a 6 well plate and incubated for 2–3 days until they reached confluence of 75-85%, after which they were trypsinised and the live cells counted using Neubauer chamber. The total number of cells at each passage was plotted on a growth curve. The procedure was repeated over 7 consecutive passages (14 days) with three biological replicas.

Apoptosis was analysed using PE Annexin V Apoptosis Detection Kit I (BD Pharmingen™, BD Biosciences) as per manufacturer’s protocol. Results were analysed by flow cytometry (BD FACS Canto II analyser) and the percentage of early apoptotic cells was determined using FACS Diva™ v6.1.3 software (BD Biosciences). Average percentage of three independent experiments was used for analysis.

### Ex vivo organotypic cerebellar slice culture

Organotypic cerebellar slices were prepared from C57BL/6 P4-P6 pups, essentially as described in [23]. The cerebellum was isolated and the meninges were carefully removed in ice cold Hank’s Balanced Solution (Sigma) supplemented with 45% glucose (Sigma) and Amphotericin B (Sigma). The cerebellum was then sagittally sectioned at 420 μm thickness using a McIlwain tissue chopper (The Mickle Lab. Engineering Co. Ltd.). The slices were kept cold for further 1 hour to prevent overt gliosis, and then 3–5 slices were placed on Millicell-CM inserts (Millipore). The inserts were transferred to Petri dish containing Modified Eagle’s Media (Sigma) and Hanks Balanced Solution (Invitrogen) supplemented with horse serum (Gibco), glutamine (Gibco), 45% glucose (Sigma) and Amphotericin B (Sigma).

To facilitate co-culture, tumour spheres were generated after harvesting cells from monolayer cell culture. For DAOY cells, 0.5-1 × 10^6^ cells were cultured in 10 ml complete media in 25 ml screw-top culture flasks and maintained at constant rotation of 70 rev/min on an orbital shaker, at 37°C until tumour spheres were obtained at 24 hr [24]. ICb1299 cells were cultured at 37°C in ultra-low cluster 6-well plate (Costar) in Dulbecco’s MEM (Invitrogen) supplemented with F12, EGF, FGF, B27 and penicillin-streptomycin until tumour spheres formed at 48 hr [25]. Tumour spheres of comparable size (at least 3–5 spheres/slice) were then seeded on the cerebellar slice cultures under stereomicroscopy and incubated for up to 8 days. The co-cultures were then fixed with 4% PFA, and stained with DAPI. Tumour cells could be identified because they were GFP positive upon lentiviral transduction and images were captured with a Confocal 710 microscope (Zeiss). Cell migration was assessed using two parameters – i) percentage of invasion area, calculated as [(total area – original tumour sphere area)/(total area) × 100], where total area is the area of migration plus that of the original tumour sphere, and ii) maximum distance of migration (distance in μm from periphery of thetumour sphere to the most distal migrated tumour cell) using Zen 2011 software (Zeiss). Three areas were assessed on each slice and a total of three slices were analysed for each experimental group. All experiments were conducted in triplicates.

### Immunocytochemistry and immunohistochemistry

Cells, cultured on Poly-lysine (PLL) coated coverslips, were fixed using 4% PFA and pre-treated with 5% Normal Goat Serum, followed by incubation with primary antibodies, either mouse monoclonal anti-BMI1 (Millipore) 1:500 or rabbit polyclonal anti-pSmad1/5/8 (Cell Signalling) 1:100. Appropriate fluorescent secondary antibodies were used, goat anti-mouse 546 (red, Invitrogen) 1:400 or goat anti-rabbit 488 (green, Invitrogen) 1:400. The coverslips were counterstained with DAPI and mounted on glass slides. Five random fluorescent micrographs (20X magnification) were obtained using a Leica DFC350 microscope. The total number of cells (DAPI positive) and the number of pSmad1/5/8 or BMI1 positive cells were counted using ImageJ software. The values were expressed as mean ± SD. The overlay pictures were used to count the clusters of cells with the same method. All experiments were carried out in triplicates.

Freshly frozen tissue sections (xenografts fixed with 4% PFA and embedded in OCT) were initially treated with cold methanol for 10 min followed by either 5% Normal Goat Serum or 10% Normal Donkey Serum for 1 hr. They were then incubated with either goat polyclonal anti-BMI1 (Santa Cruz Biotechnology) 1:100 or rabbit polyclonal anti-pSmad1/5/8 (Cell Signalling) 1:100 primary antibody overnight at room temperature. Appropriate secondary antibody was used: donkey anti-goat 568 (red, Invitrogen) 1:400 or goat anti-rabbit 546 (red, Invitrogen) 1:400 for 2 hr at room temperature. The sections were counterstained with DAPI and examined using Confocal 710 microscope (Zeiss).

For formalin fixed paraffin embedded tissue sections antigen retrieval with microwave heat treatment in citric acid monohydrate buffer of pH 6 was done. They were pre-treated with 2.5% Normal Horse Serum (Vector) for 1 hr. Primary antibodies used were: rabbit polyclonal anti-synaptophysin (DAKO) 1:200, rabbit polyclonal anti-CD44 (Abcam) 20 μl/ml, mouse monoclonal anti-Thrombospondin (Abcam) 1:25. Universal biotinylated anti-mouse/anti-rabbit IgG (Vector) secondary antibody was used. Vecstatin ABC reagent (Vector) and DAB reagent (Sigma) for 2–10 minutes was applied. All slides were counterstained by Gill’s Hematoxylin and mounted using DPX on glass cover slips.

### In vivo orthotopic xenografts

All procedures had Home Office approval (Animals Scientific Procedures Act 1986, PPL 70/7275). NOD-SCID P4-6 mice were anaesthetized according to standard procedure. Tumour cells (105 cells resuspended in 2 μl sterile PBS) were injected into the right cerebellar hemisphere (2 mm lateral and 2 mm posterior to lambda, 2 mm deep) with a 26 gauge Hamilton syringe needle. Mice were culled when developing neurological signs or at the end of the experiment (12 weeks after transplantation). The cerebellum and brain stem were harvested, fixed in 4% paraformaldehyde and cryopreserved in OCT. The entire cerebellum and brain stem were serially sectioned at 20 μm thickness and stained with DAPI. Every twelfth section was assessed for GFP positivity under fluorescence stereomicroscope (Nikon Eclipse 80i) using 10X (aperture 0.45) objective. The tumour volume, as assessed by GFP positivity, was estimated in each cerebellum by Cavalieri probe (Ghulam Muhammad et al. 2009, Villeneuve et al. 2005) using Stereo Investigator 10 software (MicroBrightField, Inc.). The grid points (20 μm grid spacing) overlapping the tumour areas were counted and were converted into volume estimates after accounting for the non-consecutive section interval and section thickness. The maximum depth of invasion (distance in μm, average of three areas) from the surface into the cerebellum, brain stem and along the Virchow Robin spaces were measured using ImageJ 1.43u software (National Institutes of Health, USA).

### Preparation, culturing and cell adhesion genes expression analysis of GCPs

Cerebella were isolated from P7 control and Bmi1-/- pups. Upon removal of meninges and blood vessels, cerebella were chopped with a mechanical tissue chopper (400 μm thicknesses), followed by digestion with trypsin in HIB buffer (120 mM NaCl, 5 mM KCl, 25 mM HEPES and 9.1 mM Glucose, pH 7.4) at 37°C for 12 min while gently shaking. One ml of trypsin stopper (0.375% Trypsin-inhibitor soybean, 0.3 mM MgSO47H2O, 0.125% DNase I) was then added to stop the reaction and the sample were quickly spun. The supernatant was discarded and the pellet was resuspended with 10 ml of pre-equilibrated culture medium (high Glucose DMEM with 10% FBS, 1% Glutamine, 1% P/S and 25 mM KCl included). The tissue was then further triturated with a 10 ml syringe and a 2 inch of 18 gauge needle for 5 times and centrifuged for 12 min at 1000 rpm. The supernatant was carefully removed and the cell pellet was resuspended in fresh medium. The clumps of cells were left to settle down for 120 s. The supernatant single cell suspension was transferred to a new 50 ml tube. Cells were seeded into 24-well (0.4 × 10^6^ cells/well) or 6-well (2.4 × 10^6^ cells/well). 0.01% PLL pre-coated coverslips were used when appropriate.

Bmi1-/- and control GCPs (n > 3), either untreated or treated with Ng (1.0 μg/μl), were harvested after 24 h. The cell pellets were lysed in RLT buffer from RNeasy Mini purification kit (Qiagen, Valencia, CA) and the lysate applied onto Qiagen shredder spin columns for homogenization. Total RNA was isolated using RNeasy purification kit and the extra On-column DNase Digestion was carried out to remove genomic DNA. cDNA synthesis was performed with RT2 First Strand Kit (SABiosciences, US). Gene expression profiles of GCPs were analysed with RT2 Profiler™ PCR Array Mouse Extracellular Matrix and Adhesion Molecules (SABiosciences, US), the manufacturer’s protocol was strictly followed. The Ct value of all the genes analysed were normalized and the difference between BMI1-/- and control samples were described by fold change. Student’s *T* test was used for statistical analysis.

### Statistical analysis

All *in vitro* and *ex vivo* experiments were performed at least in triplicates. A minimum of 6 in vivo xenograft models were used for each group for tumour volume and invasion analysis, and three xenograft tumours from each group were used for pSMAD1,5,8 expression analysis. Mean values are presented with error bars corresponding to ± SD. Statistical analysis was performed by using Prism statistical analysis software (GraphPad). Significance is indicated as ∗∗∗p < 0.001; ∗∗p < 0.01; ∗p < 0.05.

## Results

### Bmi1 dependent BMP pathway repression differentially affects the expression of selected cell adhesion genes in cerebellar granule cell progenitors

Using a genetically engineered mouse model, we recently demonstrated that cell-cell interactions between granule and glial progenitors are critically affected by Bmi1 during cerebellar development, through specific inhibition of BMP signalling [17]. As BMP signalling is known to regulate cell-cell and/or cell-extracellular matrix (ECM) interactions, thereby controlling cell motility (reviewed in [26]), we set out to analyse whether Bmi1 could regulate the expression of cell-cell and cell-matrix interaction genes in GCPs. GCPs were isolated from P7 cerebella of *Bmi1*^*-/-*^ mice and control littermates, total RNA was extracted after 1 day in culture (DIV1) and real time PCR expression arrays were used to analyse the expression of 84 genes related to cell adhesion. Eighteen (n = 18) cell-cell/matrix interaction genes were expressed at significantly higher level in *Bmi1*^*-/-*^ GCPs (p < 0.05) (Additional file 1: Table S1), of which 12 showed more than 2-fold increase in their expression level (range 2.11-5.68). These genes included Thrombospondin1, 2 and Fibronectin, Fibulin, Collagens -type I, IV, V and VI, Laminin α1 as well as CD44 and MMP 2, 8, 10.

Next, we set out to assess whether BMP pathway inhibition would affect the expression of Bmi1-regulated cell adhesion and extracellular matrix genes. Cultures were prepared from P7 cerebella of *Bmi1*^*-/-*^ and control littermates, in triplicate as previously described, and were treated with Noggin prior to expression analysis. Noggin (Ng) is a well-characterised inhibitor of BMP signalling which competitively binds BMP cell surface receptors [27]. We identified 4 Bmi1-regulated cell adhesion genes whose expression was significantly (p < 0.05) downregulated upon Noggin treatment (Additional file 1: Table S1). These genes were Thrombospondin 2, CD44, MMP10 and Collagen 6a1.

**Figure 1:**
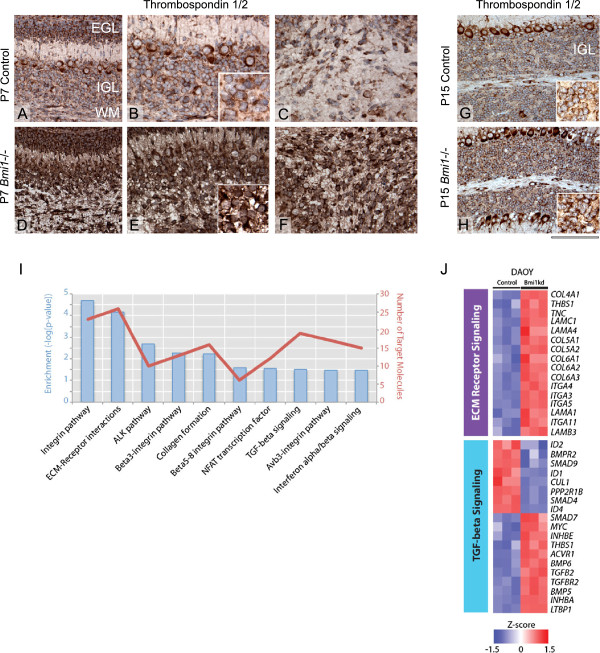
**BMI1 regulates the expression of cell adhesion genes in GCPs and ECM remodelling and TGFβ signalling in DAOY MB cells. (A-H)** Thrombospondin 1/2 immunohistochemistry on murine P7 cerebella show increased expression in GCPs in *Bmi1*-/- mice **(D and E)** compared to the control cerebellum **(A and B)**, higher magnification is shown in the inset. Increased expression of Thrombospondin 1/2 is also seen in the white matter glial cells of *Bmi1*-/- **(F)** compared to P7 controls **(C)**. Granule cells in the IGL of *Bmi1*-/- P15 mice also display increased Thrombospondin 1/2 expression **(H)** as compared to control **(G)**, inset show higher magnification. **(I)** Molecular Signature Database (MSigDB) analysis of genes differentially expressed between DAOY^BMI1kd^ versus control cells identifies significantly (FDR q < 0.05) over-represented pathways [25]. **(J)** Heat map representation of the relative upregulation of ECM receptor signalling genes (upper panel) and silencing of TGF-beta signalling (lower panel) in DAOY^BMI1kd^ compared to control cells. Genes were identified using MSigDB and plotted to highlight differences in expression of 3 z-scores or greater between the two groups (red = relative upregulation, blue = relative downregulation). EGL: External granular layer, IGL: Internal granular layer, WM: White matter. Scale bar is 125 μm **(A, D, G, H)** and 80 μm **(B, E, C, F)** and 50 μm (**B** inset, **E** inset, **G** inset, **H** inset).

In agreement with the qPCR results, widespread upregulation of Thrombospondins was observed by immunohistochemistry in GCPs (Figure 1A,D), granule cells (Figure 1A,D and B,E and G,H) as well as in white matter glial cells (Figure 1C,F and G,H) in the cerebellum of *Bmi1*^-/-^ mice at P7 and P15. We observed similar expression patterns of CD44, although the differences between mutant and controls were less prominent (Additional file 2: Figure S1A-H).

Our data suggest that Bmi1 may regulate a subset of cell adhesion genes through BMP pathway repression during cerebellar development.

### Expression of TGFβ-regulated cell adhesion molecules is controlled by BMI1 in MB

Next we set out to examine whether BMI1-mediated repression of the BMP pathway remains intact in MB. Using a publicly available transcriptome-wide analysis of DAOY MB cell line [28] we identified 1483 genes differentially expressed (>1.5 fold change, P < 0.05) between BMI1-shRNA knockdown (DAOY^BMI1kd^) and control MB cells (Additional file 3: Table S2). A Molecular Signature Databases (MSigDB) analysis identified over-represented canonical pathways that included genes related to ECM-receptor interactions as well as the TGFβ pathway (Figure 1I). Importantly among the deregulated cell adhesion molecules (ITGA3, LAMB3, LAMC1, COL7A1, Thrombospondin 1 and CD44, Figure 1J), several either represented the human homologue of the genes we had identified in *Bmi1*-/- granule cell progenitors (Additional file 1: Table S1) or belong to the same protein family.

**Figure 2:**
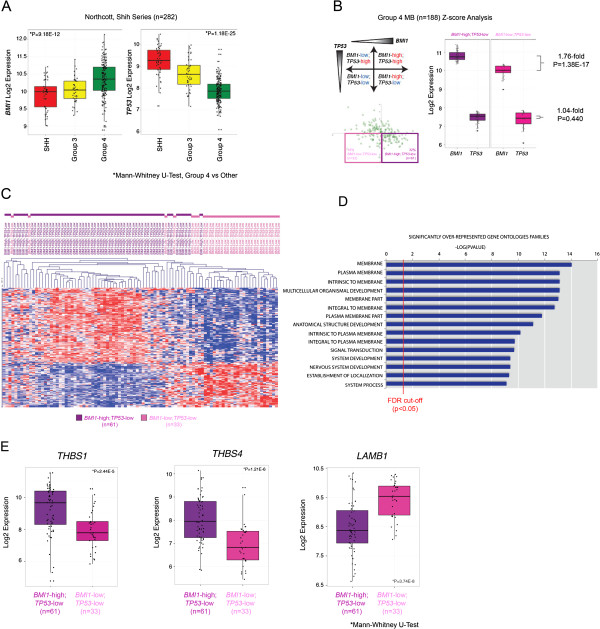
**Deregulated expression of cell adhesion molecules in BMI1-high; TP53-low Group 4 MB. (A)** Box plot representation of BMI1 expression across a cohort of 282 primary MB profiled on Affymetrix 1.1^ST^ gene arrays. *BMI1* was significantly overexpressed in Group 4 MBs versus other molecular subgroups (p = 9.18e-12). Conversely, *TP53* was significantly downregulated in Group 4 versus other molecular subgroups (p = 1.18e-25). **(B)** Z-score analysis of 282 non-WNT primary MBs revealed 32%, (61/188) of Group 4 MBs demonstrated relatively high levels of *BMI1* with concomitant reduced levels of *TP53*, whereas 18% of MBs demonstrated relatively low levels of both *BMI1* and *TP53*. Between these two populations there was a statistically significant difference in BMI1 expression, of 1.76-fold. **(C)** Unsupervised hierarchical clustering (HCL) of the top 1500 most differentially expressed genes demonstrated that these two Group 4 molecular variants (*BMI1*-high, *TP53*-low versus *BMI1*-low, *TP53*-low) largely cluster apart. **(D)** Molecular Signature Database (MSigDB) analysis of differentially expressed genes between *BMI1*-high, *TP53*-low versus *BMI1*-low, *TP53*-low Group 4 MBs identified significantly (FDR q < 0.05) over-represented Gene Ontologies (GO) intrinsic to the plasma membrane and critical for cell migration **(E)** Box plot representation of the expression of thrombospondin and laminin family members in *BMI1*-high, *TP53*-low versus *BMI1*-low, *TP53*-low Group 4 MBs identified in Bmi1-/- GCPs and human MB cell line upon BMI1 knockdown.

To further establish the connection between *BMI1* and TGFβ-regulated cell adhesion molecules identified in murine GCPs and MB cell lines we examined gene expression patterns across large cohorts of human primary MB samples (n = 282). Previously, we reported that Group 4 MBs display the highest expression of *BMI1*, relative to other molecular subgroups, while concomitantly displaying the lowest *TP53* expression [9]. Furthermore, in animal models of this disease, while *BMI1* overexpression alone is insufficient to initiate MB, BMI1 overexpression in the context of deletion of *TP53* drives MB formation [9]. Given the *BMI1*-high/*TP53*-low molecular signature associated with Group 4 MB (Figure 2A), and the resultant phenotype observed in mouse models recapitulating this genotype, we characterized the transcriptional network associated with BMI1 expression in this molecular subgroup. We identified two subgroups of Group 4 MB on the basis of *BMI1* expression levels (high versus low) (Figure 2B), while concomitantly expressing relatively low levels of TP53 to characterize the cooperative events that may contribute to MB genesis. Thirty-two percent (32%, 61/188) of Group 4 MBs analysed demonstrate relatively high levels of *BMI1* with concomitant reduced levels of *TP53*, whereas 18% of MBs demonstrate relatively low levels of both *BMI1* and *TP53*. Using unsupervised hierarchical clustering (HCL) we demonstrate that these two Group 4 molecular variants (*BMI1*-high, TP53-low versus *BMI1*-low, TP53-low) cluster apart suggesting that a distinct transcriptome-wide gene signature associate with the expression of *BMI1* (Figure 2C). A transcriptome wide analysis of *BMI1*-high, *TP53*-low versus *BMI1*-low, *TP53*-low Group 4 tumours revealed 542 genes with a statistically significant (q < 0.01) and differential (1.5-fold) expression pattern (Additional file 4: Table S3). The affected genes largely cluster into Gene Ontology (GO) families localized to the plasma membrane and involved in signal transduction, and cell-to-cell signalling (Figure 2D, Additional file 5: Table S4). Furthermore, our analysis identified some of the same cell adhesion molecules observed as differentially expressed in *Bmi1*-/- GCPs and human MB cell lines upon BMI1 knockdown, including: THBS1 (+2.44), Laminin B1 (LAMB1, -1.93, Figure 2E), EFEMP2 (+1.71), FBN2 (+2.21), SMC3 (1.53), Thrombospondin 4 (THBS4, +2.12, Figure 2E).

These data suggest that BMI1 may exert its role in human MB pathogenesis at least in part through modulation of the expression of cell adhesion genes, potentially via BMP pathway repression.

### BMI1 represses the BMP pathway in MB cell lines and in primary Group 4 MB cells

**Figure 3:**
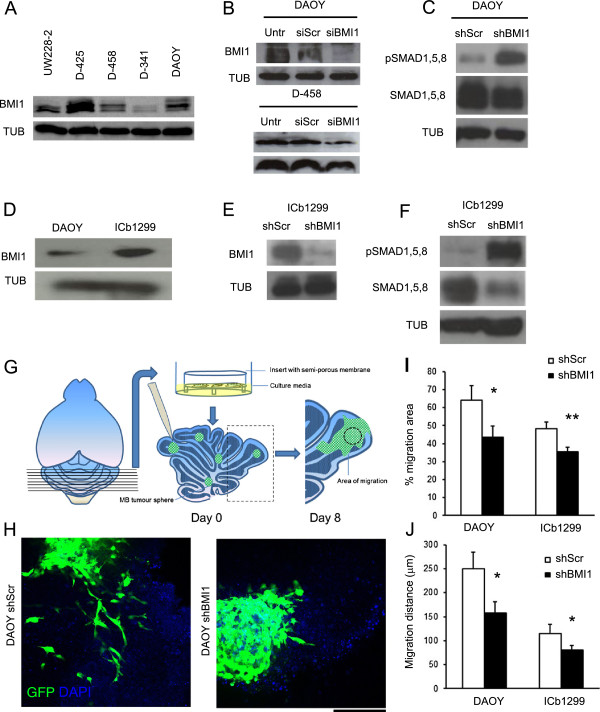
**BMI1 represses the BMP pathway and controls cell migration of primary Group 4 MB cells and MB cell lines. (A)** Analysis of BMI1 protein expression in various MB cell lines. **(B)** Effective BMI1 knock down is seen in DAOY and D-458 48 hrs after treatment with BMI1 siRNA, as compared to siScr and Untreated (Untr) controls. **(C)** Increased phosphorylation of SMAD1,5,8 proteins (pSMAD1,5,8) as compared to total SMAD1,5,8 is seen in DAOY cells following BMI1 shRNA treatment (shBMI1) as compared to shScr. **(D)** BMI1 protein expression is seen in primary Group 4 MB cells (ICb-1299), and the expression levels are comparable to that of DAOY. **(E)** An effective BMI1 knock down is achieved in ICb-1299 primary cells following BMI1 shRNA treatment. **(F)** Increased pSMAD1,5,8 expression as compared to total SMAD1,5,8 is also seen in ICb-1299 upon BMI1 knock down, indicative of BMP pathway activation. **(G)** Schematic of organotypic cerebellar slice co-culture used as an ex vivo assay to study MB cell migration. The DAOY or ICb-1299 cell spheres are placed on the slices and allowed to migrate for 8 days. **(H)** Confocal images at day 8 demonstrate migration of GFP labelled DAOY cells on the cerebellar slice. Reduced cell migration in shBMI1 (right) compared to shScr treated (left) DAOY cells. **(I and J)** Decreased area of migration **(I)** and a decreased distance of migration **(J)** is seen in both DAOY and ICb-1299 cells treated with shBMI1, compared to shScr controls. Three areas were assessed on each slice and a total of three slices were analysed in each group (N = 3). Error bars in graphs represent SD. *, P < 0.05; **, P < 0.01. Scale bar is 250 μm. Abbreviations: GFP, Green Fluorescent Protein; DAPI, 4′,6-diamidino-2-phenylindole.

*BMI1* is expressed in several MB cell lines (Figure 3A), at levels comparable to those observed in human tumour tissue samples [8]. Conditions for effective BMI1 knock down were established for two extensively characterized cell lines, DAOY and D458, with both transient lipofection-mediated siRNA delivery and stable lentiviral-mediated shRNA delivery (Figure 3B and Additional file 2: Figure S2). MB cell lines were chosen to begin our analysis because 1) they are very well characterised, extensively used, amenable to manipulation of gene expression and 2) a functional analysis in these cells would match the publicly available expression analysis dataset we have used for data mining [28].

Phosphorylation of SMAD1/5/8 is the main functional indicator of BMP pathway activation [11] and its detection is commonly used to assess pathway status. Increased phosphorylation of SMAD1/5/8 in relation to total SMAD1,5,8 was observed in DAOY^BMI1kd^ as compared to DAOY^Scr^ (Figure 3C).

Next, we used short-term cultures from a MB of Group 4, maintained as an intracerebellar xenograft, here referred to as ICb1299 [19]. We demonstrated that ICb1299 expressed BMI1 at levels comparable to those detected in DAOY (Figure 3D) and that lentiviral mediated shRNA delivery efficiently silenced BMI1 expression (Figure 3E). Furthermore, we showed increased phosphorylation of SMAD1/5/8 in relation to total SMAD1,5,8 also in these short term MB cultures upon BMI1 silencing (Figure 3F), in keeping with a scenario where BMI1 represses BMP pathway in human MB cells.

### BMI1 controls cell migration of primary MB cells in an ex vivo organotypic cerebellar slice co-culture assay

Organotypic slice cultures originally developed to study neuron-specific interactions and neuronal development of the cerebellum *in vitro*, retain some aspects of the anatomical complexity of the developing cerebellum and have been also successfully used to study and quantify invasion, proliferation and angiogenesis of established glioma cell lines [23,29].

We prepared organotypic cerebellar slices of 420 μm nominal thickness from the cerebellum of C57BL/6 P4-6 pups and cultured them on porous membranes in a chamber containing medium for a minimum of 24 hours. ICb1299 were maintained as tumour spheres in culture for few passages to amplify the culture and to effectively knock down BMI1. For the purposes of comparison, DAOY were also cultured as tumour spheres for this specific experiment. Tumour spheres of comparable size for each cell type were transferred onto the surface of viable slices and co-cultured with the slices for 8 days (Figure 3G). MB cells were identified taking advantage of the GFP labelling conferred to them by the lentiviral infection. The original tumour spheres were identified based on morphology and cell migration was assessed by analysing the maximum distance of migration from the edge of the tumour sphere and the percentage change in migration area [23]. After 8 days of co-culture, both DAOY^BMI1kd^ and ICb1299^BMI1kd^ demonstrated a reduced area of migration 43.63% (± 6.06) vs. 64.23% (± 7.83) in DAOY (p = 0.021) and 35.34% (± 2.64) vs. 48.19% (± 3.74) in ICb1299 (p = 0.008); and a reduced distance of migration as compared to control shRNA scr treated cells - 157.40 μm (± 23.38) vs. 250.03 μm (± 34.71) in DAOY (p = 0.017), and 80.50 μm (± 23.37) vs. 115.28 μm (± 34.71) in ICb1299 (p = 0.041) (Figure 3H,I,J).

These data show that the migratory properties of MB cells are influenced by BMI1 expression in both MB cell lines and in short term cultures of MB Group 4.

### Tumour volume and parenchymal invasion but not leptomeningeal spreading is controlled by BMI1 in an orthotopic MB xenograft model

**Figure 4:**
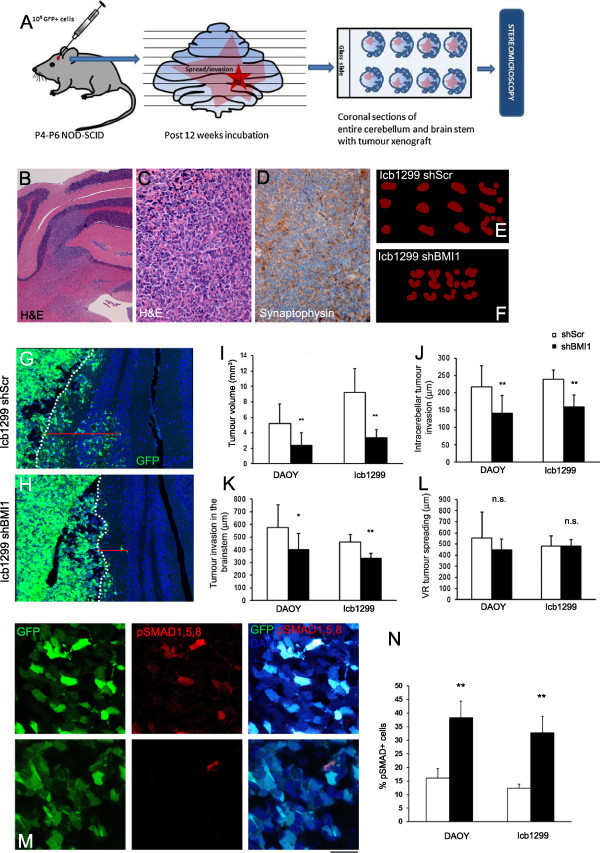
**Tumour volume and parenchymal invasion is controlled by BMI1 in an orthotopic MB xenograft model. (A)** Schematic showing generation and analysis of orthotopic MB xenografts. **(B-D)** Histology of the ICb-1299 xenografts shows a multifocal poorly differentiated tumour in the cerebellum, which show widespread Synaptophysin expression. **(E-F)** 2D representation of tumour volume estimates by Cavalieri probe using Stereo Investigator software. The overlapping tumour areas marked during Stereomicroscopic evaluation are represented in red (20 μm grid spacing used). **(G-H)** Confocal images showing a reduced intra-cerebellar invasion of GFP labelled ICb-1299 cells in shBMI1 xenograft **(H)** when compared to shScr **(G)**. The depth of invasion (μm) is measured from the deepest cell to the pial surface (dotted line) (N ≥ 6 in each group). **(I)** Quantification of tumour volume estimated by Cavalieri probe using Stereomicroscope, showing reduced volume (mm^3^) in shBMI1 group compared to shScr group in both DAOY and ICb-1299 xenografts. **(J-K)** The average distance of invasion (μm) of tumour cells from the pial surface into the cerebellar parenchyma **(J)**, and into the Brain stem **(K)**, show a reduction in shBMI1 groups in both DAOY and ICb-1299 xenografts. However, the average distance of spread of tumour cell along the Virchow Robin space is not significantly different **(L)**. The distances were quantified using acquisitions from Confocal microscopy (N ≥ 6). **(M)** pSMAD1,5,8 immunohistochemistry in ICb-1299 xenografts showing aberrant activation of BMP pathway in ICb-1299^BMI1kd^ (upper panel) as compared to ICb-1299^Scr^ (lower panel) **(N)** Quantification of percentage of pSMAD1,5,8 expressing cells in selected xenografts (N = 3). Scale bar 1 mm **(B)**, 125 μm **(C, D, G, H)** and 50 μm **(M)**. Error bars in graphs represent SD. *, P < 0.05; **, P < 0.01. Abbreviations: H&E, Hematoxylin and Eosin; GFP, Green Fluorescent Protein; DAPI, 4′,6-diamidino-2-phenylindole.

To determine the contribution of BMI1 to tumour growth and invasive characteristics, DAOY^BMI1kd^ and ICb1299^BMI1kd^ as well as their control counterparts were transplanted into the cerebellum of P4-6 NOD-SCID pups. Twelve weeks after transplantation, mice were sacrificed and the cerebellum, brain stem and spinal cord were analysed histologically (Figure 4A). Histological examination identified multifocal tumour growth composed of poorly differentiated neoplastic cells with densely packed round to oval cells with hyperchromatic nuclei surrounded by scanty cytoplasm (Figure 4B,C) and diffuse expression of synaptophysin (Figure 4D). Immunohistochemical analysis confirmed prominent reduction of BMI1 expression in tumours arising from DAOY^BMIkd^ and ICb1299^BMI1kd^ cells as compared to those arising from scrambled treated cells (Additional file 2: Figure S3). 100% (18/18 mice) of mice injected with DAOY cells either DAOY^BMIkd^ or DAOY^Scr^ developed intracerebellar xenografts, while 63.2% (12/19 mice) of mice injected with ICb1299 cells developed tumours. No significant difference in tumour engraftment was observed between ICb1299^Scr^ and ICb1299^BMI1kd^ injected mice (60% vs. 66.6% respectively). Interestingly, however, estimation of the tumour volume by Cavalieri probe using Stereo Investigator software (Figure 4E,F) revealed reduced total tumour volume in mice engrafted with DAOY^BMI1kd^ cells compared to those engrafted with DAOY^Scr^ cells - 2.39 mm^3^ (± 1.63) vs. 5.18 mm^3^ (± 2.57), p = 0.009, n = 9 in each category (Figure 4I) and similar findings were observed in ICb1299^BMI1kd^ xenografts as compared to ICb1299^Scr^ - 3.35 mm^3^ (± 1.05) vs. 9.24 mm^3^ (± 3.09), p = 0.001, n = 6 in each category (Figure 4I).

Furthermore, assessment of the depth of invasion into the cerebellar parenchyma from the pial surface revealed a significant reduction for both DAOY^BMI1kd^ and ICb1299^BMI1kd^ xenografts - 141.35 μm (± 51.51) vs. 216.61 μm (± 61.24) for DAOY (p = 0.008), and 159.74 μm (± 34.96) vs. 239.49 μm (± 25.75) for ICb-1299 (p = 0.001) (Figure 4G,H and J). Similar findings were recorded when measuring depth of tumour cell invasion into the brain stem [401.78 μm (± 126.41) DAOY^BMI1kd^ vs. 575.83 μm (± 175.91) DAOY^Scr^ (p = 0.018)] and 332.78 μm (± 39.23) ICb1299^BMI1kd^ vs. 459.09 μm (± 62.06) ICb1299^Scr^ (p = 0.001) (Figure 4K). Instead, invasion along the (perivascular) Virchow Robin spaces (Figure 4L) and the leptomeningeal spread (data not shown) were not affected.

To determine the BMP pathway status in the xenografts, we performed pSMAD1,5,8 immunohistochemical labelling on DAOY^BMI1kd^, DAOY^Scr^, ICb1299^BMI1kd^ and ICb1299^Scr^ tumours. The number of MB cells expressing pSMAD1,5,8 was increased in BMI1 silenced xenografts - 38.27% (±6.16) vs. 16.02% (± 3.51) in DAOY (p = 0.005), and 32.77% (±6.0) vs. 12.33% (±1.50) in ICb-1299 (p = 0.004) (Figure 4M,N).

These observations show that BMI1 controls both tumour size and parenchymal invasion in MB xenografts and confirm that it represses BMP pathway activation also *in vivo*.

### Cell migration of MB cell lines is regulated by BMI1 in a BMP pathway dependent fashion in vitro

**Figure 5:**
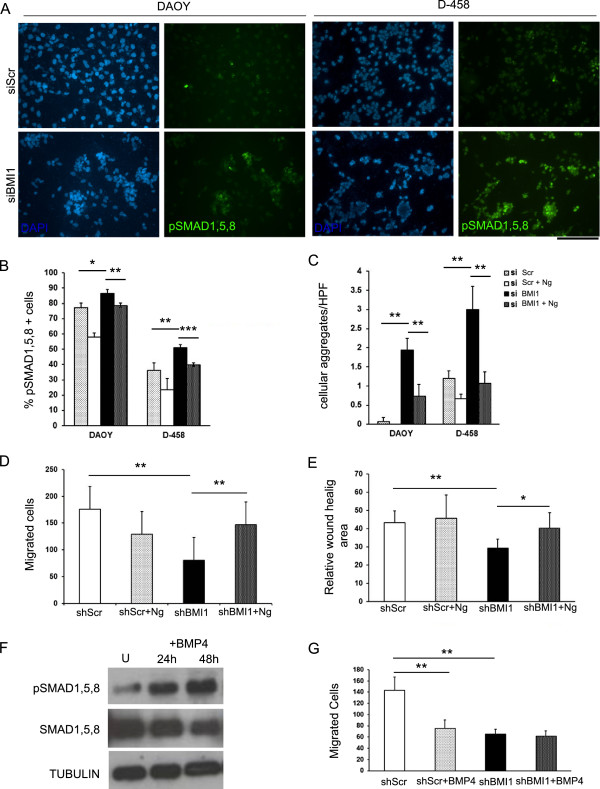
**Cell migration of MB cell lines is regulated by BMI1 in a BMP pathway dependent fashion in vitro. (A)** DAOY and D-458 cultures show an increased number of cells (in %) expressing pSMAD1,5,8 and an increased number of cohesive cell clusters upon siRNA BMI1 treatment (bottom panel) compared to siRNA Scr treatment (top panel). **(B)** Quantification of the number of cells expressing pSMAD1,5,8 in cultures with or without Noggin treatment (N = 3). **(C)** Quantification of cohesive cell clusters per HPF with or without Noggin treatment (N = 3). **(D)** Transwell migration assay shows a decreased migration of DAOY cells in shRNA BMI1 cultures compared to shRNA Scr, but when shBMI1 cultures are concomitantly treated with Noggin, cell migration is rescued (N = 3). **(E)** Gap closure assay shows similar findings. **(F)** Western blot demonstrating BMP pathway activation by means of increased pSMAD1,5,8 expression as compared to total SMAD1,5,8 in DAOY treated with BMP-4, compared to untreated cells. **(G)** Transwell migration assay shows decreased migration of DAOY cells upon BMP-4 treatment similarly to shRNA BMI1 knock down but no additive effect is seen when BMP4 treatment is applied concomitantly to shRNA BMI1 knock down (N = 4). Scale bar = 250 μm. Error bars in graphs represent SD. *, P < 0.05; **, P < 0.01. Abbreviations: DAPI, 4′,6-diamidino-2-phenylindole.

The invasiveness of malignant cells has been linked to their adhesive properties [30], raising the possibility that the reduced migration and invasion observed upon BMI1 knock down could be due to BMP-regulated changes in cell adhesion. To test this hypothesis, we used a modified Transwell Migration Assay and an *in vitro* Gap Closure Migration Assay. In support of our organotypic culture experimental results, we observed a trend to form cohesive cell clusters in both DAOY and D-458 cell lines when cultured *in vitro* upon BMI1 silencing. Quantification of the number of multicellular aggregates, as defined by cohesive clusters of 10 or more cells per 20x field, confirmed the morphological observation that BMI1 knockdown significantly increased the number of multicellular aggregates in both MB cell lines - 1.93 (+/- 0.31) vs. 0.07 (+/- 0.12) in DAOY (p = 0.004), and 3 (+/- 0.6) vs. 1.2 (+/- 0.2) in D-458 (p = 0.003) (Figure 5A,C).

Quantification of the number of pSMAD1/5/8 positive cells in DAOY^BMI1kd^ and D-458^BMI1kd^ cultures confirmed a significant increase in the number of positive cells in both cell lines upon BMI1 knock down - 86.63% (+/- 2.41) vs. 77.05% (+/- 3.25) in DAOY (p = 0.017) and 51.17% (+/- 1.74) vs. 36.06% (+/- 5.19) in D-458 (p = 0.004) (Figure 5A,B), in keeping with previous Western blot results (Figure 3C). Treatment of DAOY and D-458 cultures with Ng revealed a significant reduction of the number of pSMAD1/5/8 positive cells - 57.88% (± 2.85) vs. 77.05% (± 3.25) in DAOY (p = 0.0007) and 23.69% (± 7.19) vs. 36.06% (± 5.19) in D-458 (p = 0.036) (Figure 5B), confirming the inhibitory role of Ng on BMP pathway also in MB cell lines. When Noggin treatment was applied to DAOY^BMI1kd^ and D-458^BMI1kd^ cultures, the number of pSMAD1/5/8 positive cells was also reduced – 78.47% (± 1.35) vs. 83.63% (± 2.40) for DAOY (p = 0.006) and 39.66% (± 1.35) vs. 51.17 (± 1.74) for D-458 (p = 0.0004) (Figure 5B). Under these culturing conditions, a significant decrease in the number of cell aggregates was observed for both DAOY and D-458 - 0.73 (+/- 0.30) vs. 1.93 (+/- 0.31) in DAOY (p = 0.004), and 1.07 (+/- 0.30) vs. 3 (+/- 0.6) in D-458 (p = 0.003) (Figure 5C).

In the Transwell Migration Assay, MB cells cultured in serum-free medium were plated on the top surface of a substrate coated Transwell membrane (containing 8 μm diameter pores), while medium containing 10% serum was added to the bottom well as chemo-attractant. After incubation for 12 h, the number of cells that migrated through substrate and membrane were stained with Haematoxylin and counted. Two different adhesion substrates were used in separate experiments - matrigel (a basement membrane extract rich in extracellular matrix laminin) and type I collagen. These substrates were chosen to mimic the *in vivo* leptomeningeal environment, which mainly comprises laminin and type I collagen in the matrix structure. DAOY cells adhered well on these substrates and could be assayed while D-458 cells did not adhere and were not used for this experiment.

Whilst there was no significant difference in cell migration between the DAOY^BMI1kd^ and DAOY^Scr^ on matrigel substrate (data not shown), we observed a significant reduction of migrating cells in DAOY^BMI1kd^ cultures, compared to DAOY^Scr^ on collagen type I substrate - 80.67 (+/- 55.51) vs. 176.07 (+/- 42.38), p = 0.005 (Figure 5D), raising the possibility that Collagen type 1, which is known to be expressed in the leptomeninges [31], represent a more appropriate substrate for MB cell invasion. Importantly, decreased migration of DAOY^BMI1kd^ cells was dependent on aberrant activation of BMP pathway, as the number of migrating cells significantly increased upon Noggin treatment of DAOY^BMI1kd^ cultures - 147.23 (+/- 46.63) vs. 80.67 (+/- 55.51), p = 0.004 (Figure 5D). No significant difference in cell migration was noted upon Noggin treatment of DAOY^Scr^ 129.58 (+/- 72.56) vs. 176.07 (+/- 42.38), p = 0.081 (Figure 5D).

To validate the findings with an independent migration assay, DAOY cells were plated with optimum cell density and an 800 μm wide linear gap was incited. The area of gap closure was analysed using time lapse videomicroscopy over 12 hr. A significant reduction in the gap closure area was observed in the DAOY^BMI1kd^ cultures as compared to DAOY^Scr^ cultures - 29.08% (+/- 5.19) vs. 43.11% (+/- 6.47), p = 0.0025, an effect that was reverted by additional treatment with Noggin - 40.18% (+/- 8.42) and 29.08% (+/- 5.19) respectively, p = 0.048 (Figure 5E). No significant difference in gap closure was noted upon Noggin treatment of DAOY^Scr -^ 45.79% (+/- 12.59) vs. 43.11% (+/- 6.47), p = 0.12 (Figure 5E).

Next, we asked whether the changes in cluster formation and in cell migration/wound healing upon BMI1 downregulation could be influenced by the Ink4a-mediated cell cycle control exerted by BMI1 in various physiological and cancer-related contexts. In keeping with existing literature [28], we show that BMI1 downregulation significantly reduced proliferation of the DAOY cells, as assessed by two independent methods, the CyQuant fluorescence emission 280.55 ± 43.6 vs. 532.44 ± 51.6 units (p = 0.003) and the growth curve analysis (Additional file 2: Figure S4A and C). However, concomitant treatment of DAOY^BMI1kd^ with Ng did not rescue the proliferation defect (Additional file 2: Figure S4A and C) and no significant impact on apoptosis was noted upon Noggin treatment of DAOY^BMI1kd^ as assessed by Annexin V staining and FACS analysis [DAOY^BMI1kd + Ng^ vs. DAOY^BMIkd^ = 78.58% (+/- 10.77) vs. 80.13% (+/- 11.15), p = 0.434] (Additional file 2: Figure S4B).

Taken together these results support the conclusion that (i) BMI1-mediated control of proliferation is BMP-independent and (ii) BMI1/BMP mediated control of cell adhesion and migration is independent from the well-known effect of BMI1 on cellular proliferation. In keeping with this interpretation, single cell motility tracking by time lapse microscopy confirmed reduced motility in DAOY cells upon BMI1 knock down - 8.43 μm (+/- 1.61) vs. 11.41 μm (+/- 1.69), p = 0.005 (Additional file 2: Figure S5A-C)

### BMP treatment of a MB cell line reduces cell migration in a similar fashion to BMI1 knock down and no additive effect is seen when BMP is applied after BMI1 knock down

We reasoned that BMI1-mediated repression of BMP pathway could be the molecular mechanism which is counteracted by treatment of MB cells with BMP. This treatment has been shown to be effective on MB cell lines both in vitro and in vivo, in mouse models [13]. DAOY cells were treated with BMP4 (100 ng/ml concentrations) and protein expression analysis for pSMAD1,5,8 in relation to SMAD1,5,8 demonstrated best pathway activation between 24 h and 48 h after treatment (Figure 5F). This timeframe was well within what required for the Transwell Migration Assay, which was carried out on DAOY^Scr^ and DAOY^BMI1kd^ treated with BMP4 as compared to untreated controls. As observed previously, reduction in migration was observed in DAOY^BMI1kd^ as compared to DAOY^Scr^ cultures – 65 (+/- 8.85) vs. 142.85 (+/- 24.26), p = 0.001 (Figure 5G). Whilst a significant reduction in cell migration was noted in DAOY^Scr^ treated with BMP4 as compared to untreated cells - 75.8 (+/- 14.78) vs. 142.85 (+/- 24.26), p = 0.003 (Figure 5G), no additional reduction of cell migration was seen in DAOY^BMI1kd^ cultures treated with BMP4 as compared to DAOY^BMI1kd^ without BMP4 treatment - 61.84 (+/- 9.07) vs. 65 (+/- 8.85), p = 0.160 (Figure 5G).

These findings raise the possibility that expression of BMI1 could represent a biomarker for MB which could benefit from a treatment with small molecules acting as BMP agonists.

## Discussion

Bmi1 plays an important role in the postnatal development of the cerebellum and its deficiency leads to developmental defects affecting both the neuronal and glial lineages in mice [8,17]. The best characterized function of Bmi1 is the control of proliferation of undifferentiated progenitor cells mainly through repression of the Ink4a/Arf tumour suppressor gene locus [32,33], which in turn regulates the activity of cyclin D, Cdk4/Cdk6 and p53 (reviewed in [34]). BMI1 is overexpressed in a significant proportion of MB affecting a multitude of cellular processes, of which SHH-driven MB proliferation has been most extensively interrogated [8,35]. However, we have recently reported that BMI1 also regulates cell adhesion and migration of cerebellar progenitors through repression of the BMP pathway [17]. These findings are in keeping with chromatin immunoprecipitation coupled with microarray experiments which have shown BMPs to be direct targets of BMI1 in fibroblasts [36] and also with the results of a recent paper showing that fine-tuning of the expression of direct effectors or inhibitors of the BMP pathway, such as for examples Id1-Atf3, by BMI1 occurs in adult neural progenitor cells [37].

BMPs are members of the TGFβ signalling pathway and their role during cerebellar development and in MB pathogenesis is well characterized. BMP2 and BMP4 favour the switch from proliferation to differentiation of GCPs by antagonizing the mitogen SHH and similarly induce an anti-proliferative role in murine MB cells [13]. BMP-mediated regulation of cell adhesion and of the cellular interactions with the extracellular matrix have been demonstrated also in other cellular contexts such as for example in soft tissues remodelling [38].

Here, we provide evidence that BMI1 controls tumour volume and intraparenchymal invasion in an orthotopic xenograft model of MB. While the reduced tumour volume observed upon BMI1 silencing follows previous reports where reduced tumour growth was seen in subcutaneous DAOY xenografts upon shRNA BMI1 knock down [28], the effect on brain invasion is novel. Re-analysis of a publicly available genome wide expression dataset of BMI-1 knock-down MB cell lines revealed deregulation of TGFβ pathway and differential expression of several cell adhesion molecules. Aberrant activation of BMP pathway in BMI1 silenced cells was confirmed in our xenografts. These data together with the results of the migration assays *in vitro* which show that cell adhesion and motility are controlled by BMI1 through BMP pathway inhibition, raise the possibility that this mechanism underpins also the phenotype *in vivo*. Downregulation of BMI1 expression reduces proliferation of MB cells and it is likely to contribute to the reduced tumour volume observed in our xenografts of DAOY^BMI1kd^ cells. However, we show that BMI1-mediated control of proliferation is BMP-independent and it is therefore unlikely to be responsible for the effect on motility and invasion.

Overexpression of BMI1 is found preferentially in human MB of Group 4 and overexpression of Bmi1 induces MB formation only in the context of Tp53 deletion in the mouse, albeit at very low frequency [9]. We previously reported that Group 4 MBs also display the lowest TP53 expression [9], although the mechanism for this is currently unknown, as genetic mutations of TP53 are more frequent in other subgroups. It is conceivable, however, that other mechanisms including epigenetic regulation, which incidentally is more often deregulated in Group 4 MBs [39], could be involved here and indeed low Tp53 levels may play a functionally very relevant role also in Group 4 MBs. A biostatistical analysis of human Group 4 MB overexpressing BMI1 while concomitantly expressing low levels of TP53 revealed a set of differentially expressed genes, which affected extracellular matrix structural constituents. Among these genes, members of the Thrombospondin and Laminin families were detected, which were deregulated also in DAOY^BMI1kd^ and in GCPs lacking Bmi1 in a BMP dependent fashion. GCPs and cerebellar neural stem cells (cNSC) have been shown to act as cell of origin of MB [40,41], in particular SHH group MB originates from GCPs [42]. Little is known about the cell origin of MB Group 4 but their origin from GCPs is a distinct possibility as they could have lost SHH dependency during their oncogenic transformation pathway. It will be important to improve our mouse model of MB Group 4 [9], for example with a conditional approach to selectively inactivate TPp53 in the granule cell lineage and to compare it with the human counterpart to validate or dispute this theory. Alternatively, BMI1-mediated repression of BMP could be a molecular feature of MB overexpressing BMI1, independent of molecular subgroup affiliation and cell of origin.

We show significant deregulation of extracellular matrix gene expression in human MB overexpressing BMI1. Among these genes, members of the Thrombospondin, Laminin and Collagen families were regulated by BMI1 in MB cell lines and in GCPs, in the latter case in a BMP-dependent fashion. Thrombospondins are strongly expressed in postmitotic premigratory GCPs [43] where they bind to integrins, which are involved in the control of GCPs proliferation in cooperation with SHH, as shown in mice lacking integrin β1 [44]. Interestingly type IV collagens induce expression of thrombospondins and the role of these matrix proteins in regulation of differentiation of CNS progenitors has been demonstrated [45]. Members of both the thrombospondin and [46] and collagen families [4] are deregulated in human MB with an aggressive phenotype. Taken together these data raise the possibility that invasion of MB cells is regulated by BMI1 through BMP-mediated control of cell adhesion. Interestingly we did not see increased spreading of MB cells along VR spaces in our xenograft model and tumours expressing high levels of BMI1 were not associated with higher incidence of spinal metastasis in human MB (data not shown), therefore implying that the molecular mechanisms regulating intraparenchymal invasion and leptomeningeal spread may be different.

Treatment of brain tumour stem cells isolated from glioblastoma patients with BMP reduced their tumourigenic potential through inhibition of the proliferation capacity and increased glial differentiation [47] and proliferation arrest by BMPs has been shown also for MB [13], raising the possibility that small molecules acting as BMP agonists could be developed to be used therapeutically in MB patients. Importantly, we show that the impact of BMP treatment on the invasive properties of MB cells is most effective when BMI1 is expressed at high levels, raising the possibility that BMI1 could be used as a biomarker to identify groups of patients who can benefit from a treatment with BMP agonists.

## Conclusions

In this study, we used a novel xenograft model of Group 4 MB and in vitro assays to demonstrate that BMP pathway activation is regulated by BMI1 in MB and controls cell migration and invasion potentially by regulation of extracellular matrix proteins.

## Competing interests

The authors declare that they have no competing interests.

## Authors’ contributions

AM: designed and executed *in vitro*, *ex vivo* and *in vivo* xenograft experiments including functional and immunofluorescence studies, data analysis and interpretation, and wrote the manuscript. AMD and MR: designed and executed MB gene expression profiling including data interpretation and manuscript writing. XZ: performed gene expression analysis on GCPs and immunohistochemistry on the murine cerebellum. PAB and XL: provided Group 4 primary MB cells isolated, and characterised in their laboratory. MDT: supervised human MB gene expression profiling and pathway analysis and provided expertise for the manuscript’s content. SM: conceived, designed and supervised the overall study, including interpretation and analysis of the data, wrote the manuscript and provided financial support. All authors read and approved the final manuscript.

## Supplementary Material

Additional file 1: Table S1 List of cell-cell/matrix interaction genes expressed at significantly higher level in BMI1-/- GCPs (p < 0.05), of which 12 showed more than 2-fold increase in their expression level (range 2.11-5.68). Click here for file

Additional file 2: Figure S1 Increased CD44 expression is seen in GCPs in *Bmi1*-/- P7 cerebellum **(C)** compared to the control cerebellum **(A)** [**(B)** and **(D)** are high power view of **(A)** and **(C)** respectively, and in *Bmi1-/-* P15 cerebellum **(G)** compared to control **(E)** [**(F)** and **(H)** are high power views of **(E)** and **(G)** respectively]. Scale bar = 250 μm. **Figure S2.** Efficient BMI1 knock down in DAOY cells upon lentiviral mediated shRNA treatment. **Figure S3.** BMI1 immunohistochemistry on xenografts reveals an effective BMI1 knock down in vivo. **Figure S4.** CyQuant fluorescence dye binding assay **(A)**, and growth curve analysis **(C)** show significantly reduced proliferation in BMI1 knock down cells compared to scrambled controls, but no significant changes in proliferation when Noggin was concomitantly added. No change in apoptosis was observed **(B)**. **Figure S5.** Time lapse experiment tracking individual cells shows decreased cell motility following BMI1 knock down in DAOY. Click here for file

Additional file 3: Table S2 List of genes differentially expressed (>1.5 fold change, P < 0.05) between BMI1- shRNA knockdown (DAOY^BMI1kd^) and control MB cells (Wiederschain et al. [28]). Click here for file

Additional file 4: Table S3 List of genes with statistically significant (P < 0.05) differential (> = 1.5-fold) expression between BMI1-high, TP53-low and BMI1-low, TP53-low group 4 tumours. Click here for file

Additional file 5: Table S4 Summary of all Gene Ontologies significantly (FDR q < 0.05) enriched in Bmi1-high, TP53-low vs. BMi1-low, TP53-low samples. Click here for file
