# Supplementary figures and images for: Polycomb group gene BMI1 controls invasion of medulloblastoma cells and inhibits BMP-regulated cell adhesion
Source: Acta Neuropathol Commun. 2014 Jan 24;2:10. doi: 10.1186/2051-5960-2-10 (PMC3928978; doi:10.1186/2051-5960-2-10)

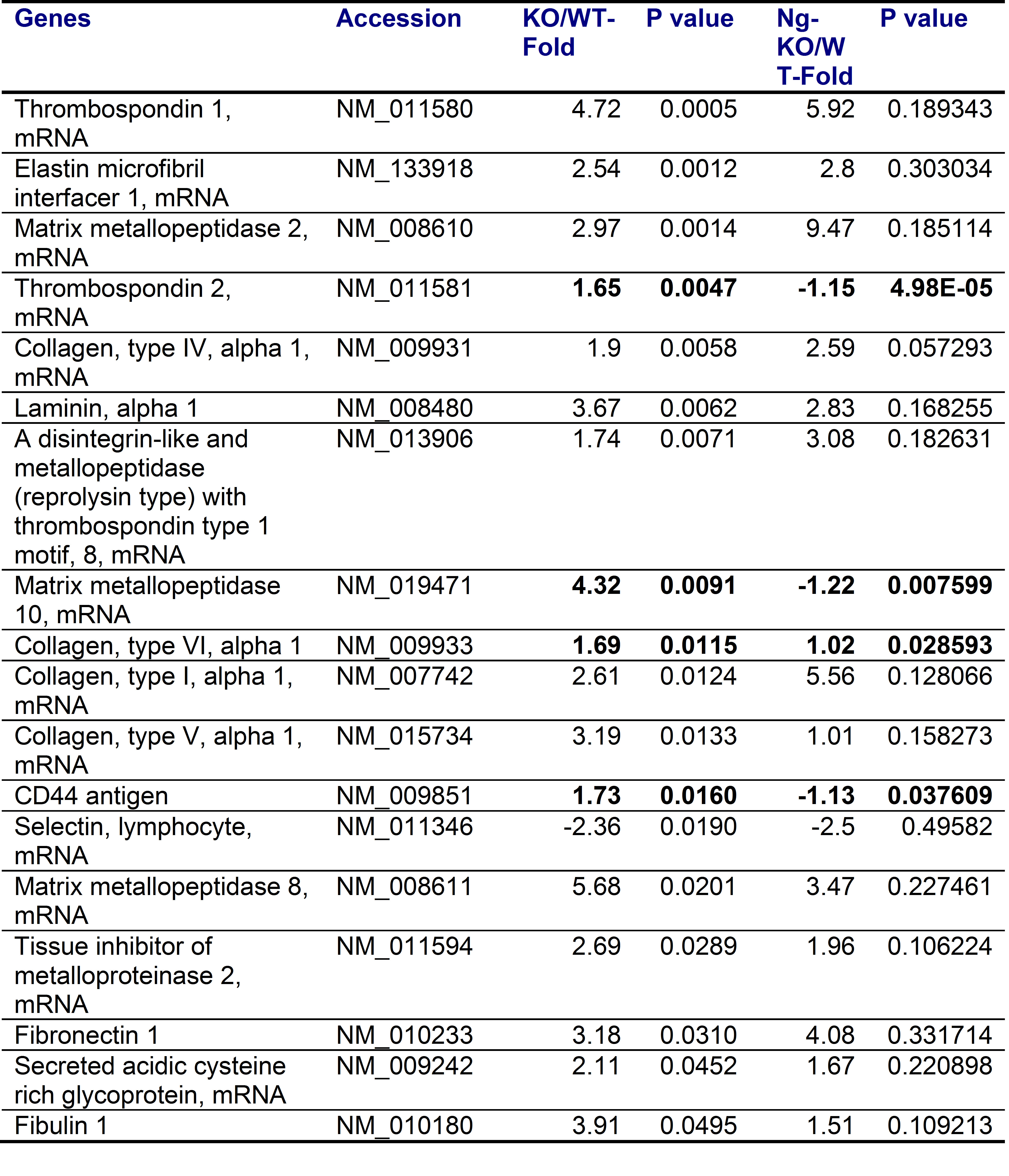

Supplement: Additional file 1: Table S1 — List of cell-cell/matrix interaction genes expressed at significantly higher level in BMI1-/- GCPs (p < 0.05), of which 12 showed more than 2-fold increase in their expression level (range 2.11-5.68). [file 2051-5960-2-10-S1.jpeg]

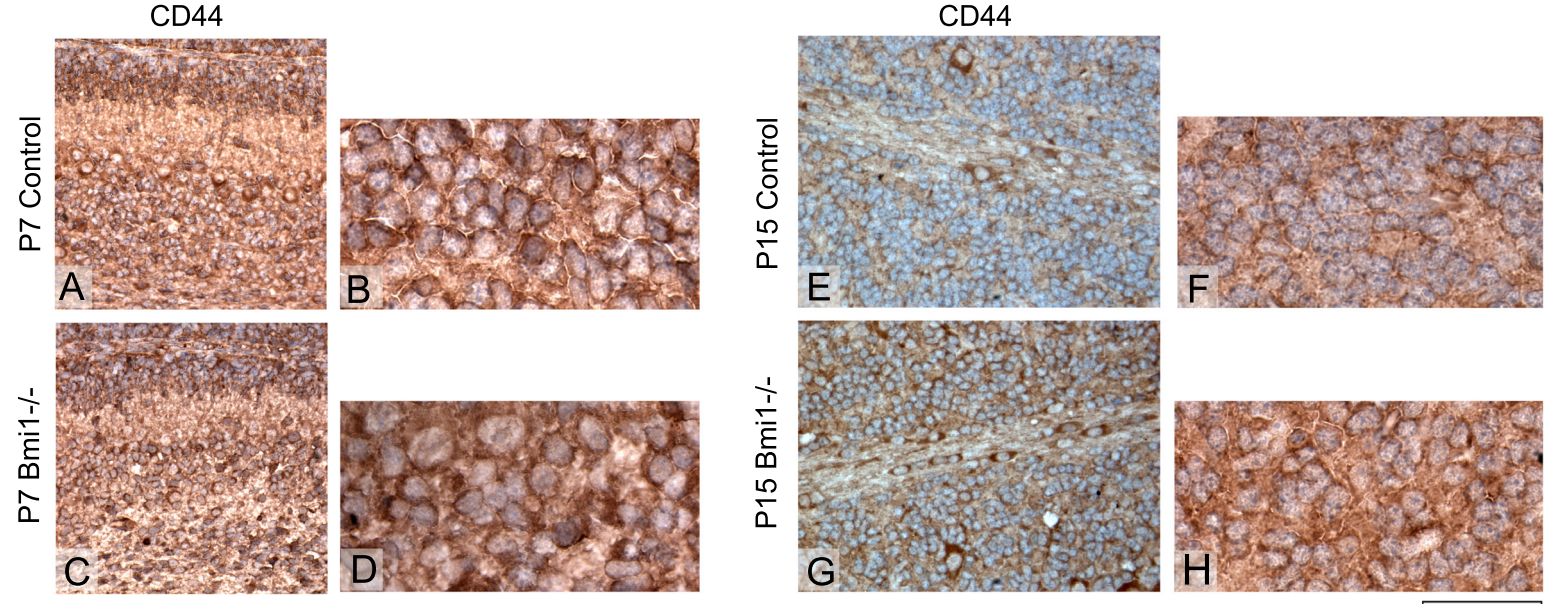

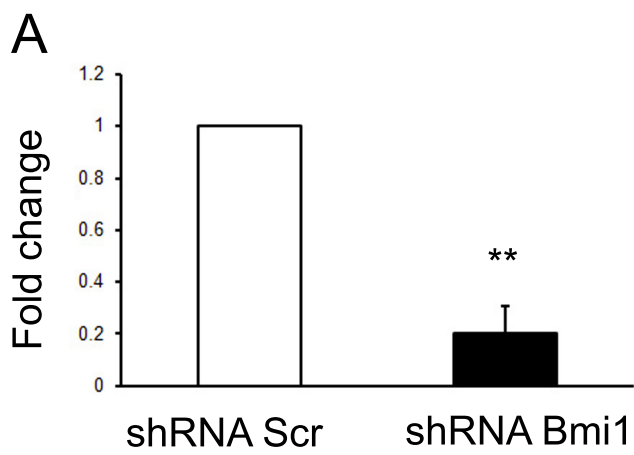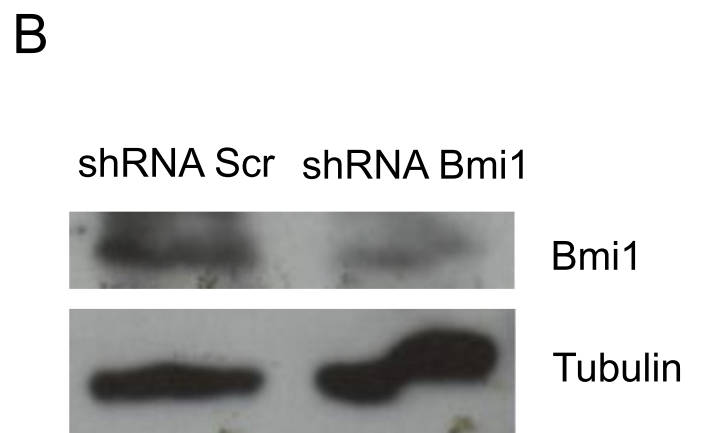

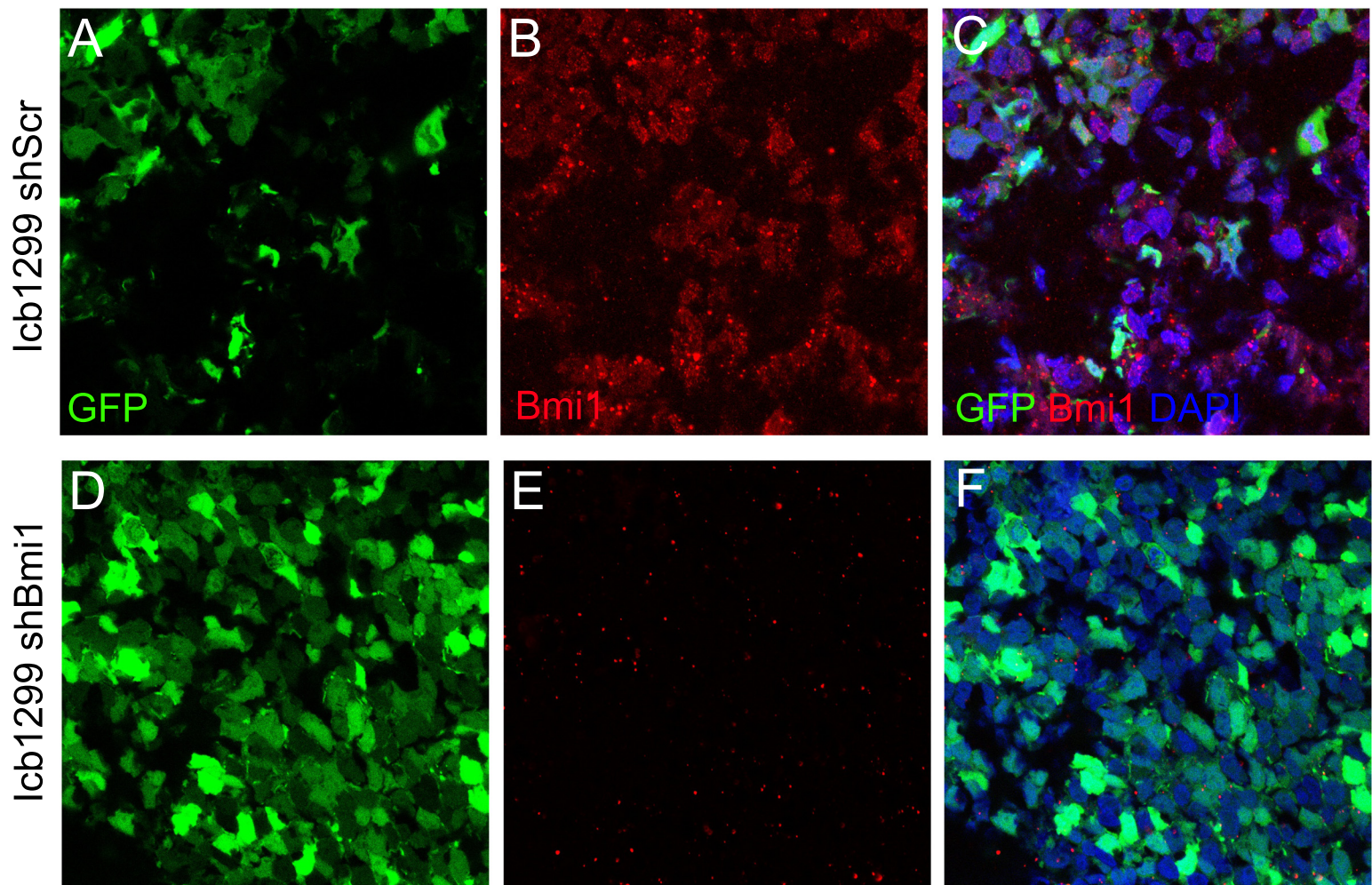

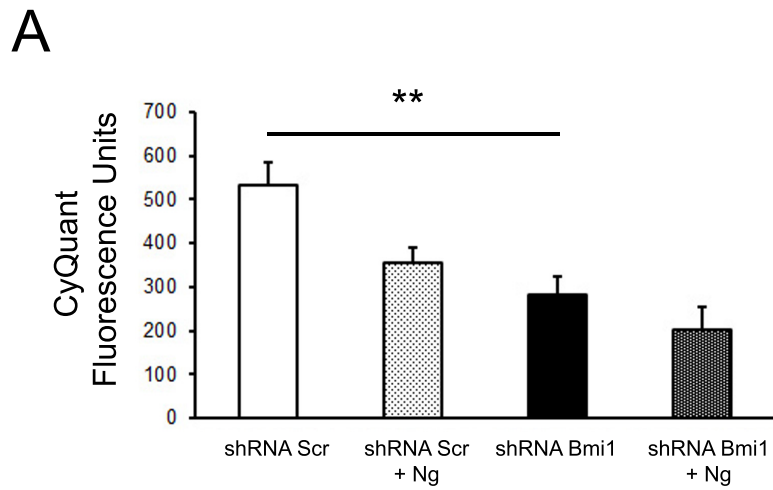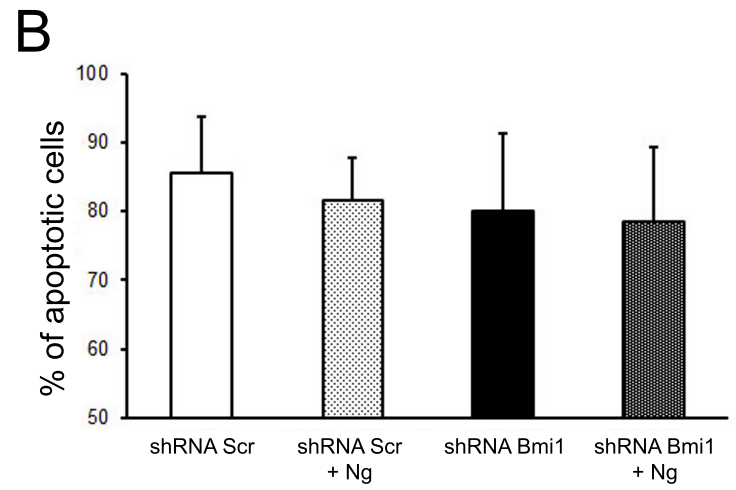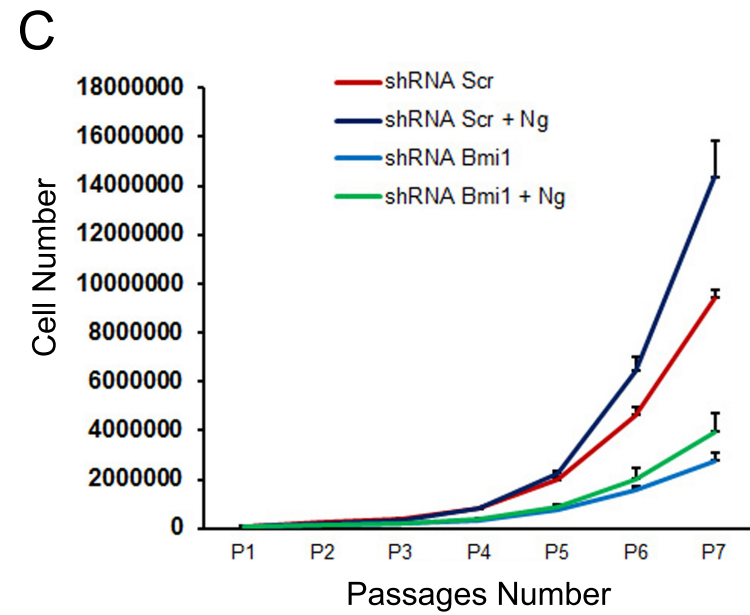

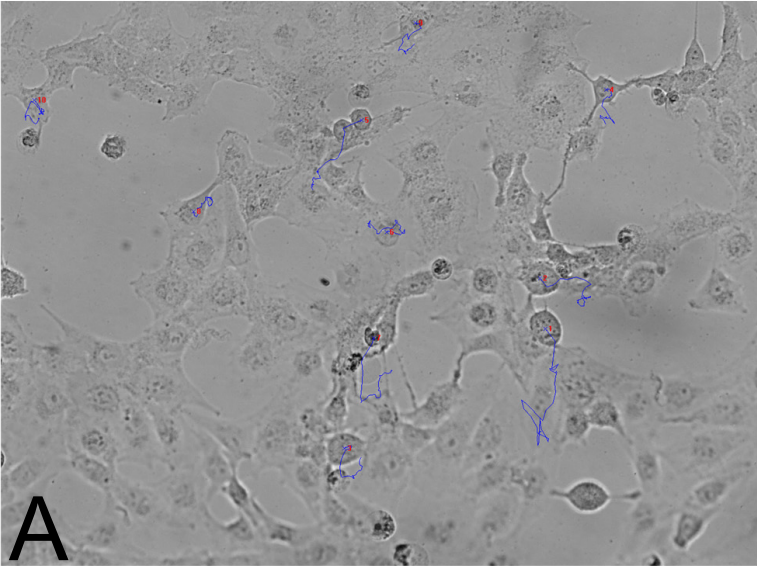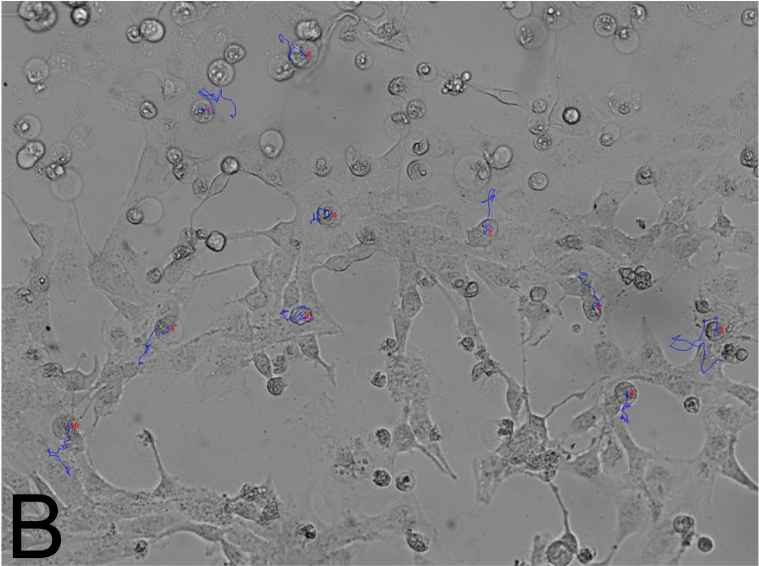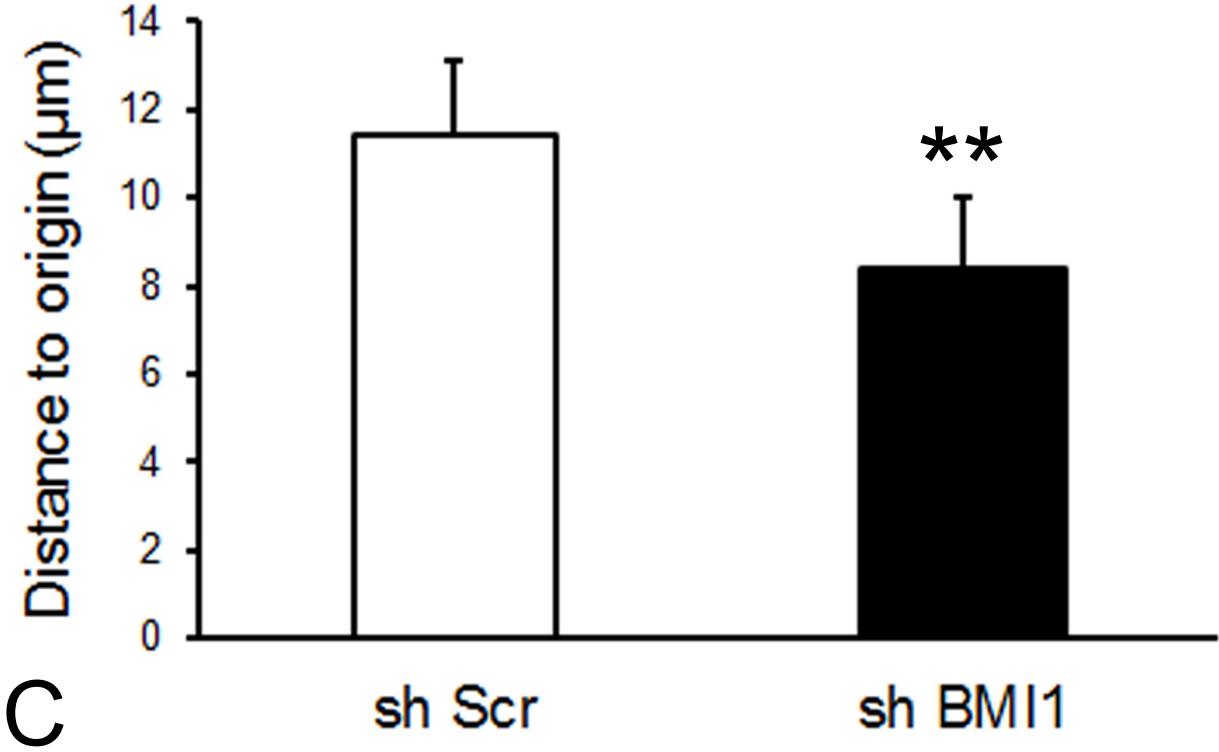

Supplement: Additional file 2: Figure S1 — Increased CD44 expression is seen in GCPs in Bmi1-/- P7 cerebellum (C) compared to the control cerebellum (A) [(B) and (D) are high power view of (A) and (C) respectively, and in Bmi1-/- P15 cerebellum (G) compared to control (E) [(F) and (H) are high power views of (E) and (G) respectively]. Scale bar = 250 μm. Figure S2. Efficient BMI1 knock down in DAOY cells upon lentiviral mediated shRNA treatment. Figure S3. BMI1 immunohistochemistry on xenografts reveals an effective BMI1 knock down in vivo. Figure S4. CyQuant fluorescence dye binding assay (A), and growth curve analysis (C) show significantly reduced proliferation in BMI1 knock down cells compared to scrambled controls, but no significant changes in proliferation when Noggin was concomitantly added. No change in apoptosis was observed (B). Figure S5. Time lapse experiment tracking individual cells shows decreased cell motility following BMI1 knock down in DAOY. [file 2051-5960-2-10-S2.pdf]
